# Low-Temperature Fabrication of Carbon Nanotube–Aluminum Composite Powders via Rotary Chemical Vapor Deposition: Process Optimization and Growth Mechanisms

**DOI:** 10.3390/ma18071654

**Published:** 2025-04-03

**Authors:** Ruodi Tan, Haifeng Li, Jianwu Liu, Zizhao Wu, Qun Wang, Chidambaram Seshadri Ramachandran

**Affiliations:** 1 College of Materials Science and Engineering, Hunan University, Changsha 410082, China; tanruodi@163.com; 2 AECC Hunan Aviation Powerplant Research Institute, Zhuzhou 412002, China; lihaifeng3737@163.com (H.L.); jianwuliu8678@163.com (J.L.); as02071731@163.com (Z.W.); 3 Department of Materials Science and Engineering, The State University of New York (SUNY) Stony Brook, New York, NY 11794, USA; csrcn1@gmail.com

**Keywords:** R-CVD, CNTs, aluminum, temperature, composite material

## Abstract

This study successfully achieved the in situ synthesis of carbon nanotubes (CNTs) on aluminum powder substrates through rotating chemical vapor deposition (R-CVD) using nickel-based catalysts with acetylene as the carbon source. Through systematic parameter optimization, we elucidated the effects of catalyst loading, synthesis temperature, reaction duration, reactor rotation speed, and carrier gas ratio on the morphology, crystallinity, and yield of CNTs. Comprehensive characterization employing transmission electron microscopy (TEM), scanning electron microscopy (SEM), Raman spectroscopy, and X-ray diffraction (XRD) demonstrated that R-CVD enables low-temperature synthesis (480 °C) of CNTs with enhanced crystallinity, improved yield, and uniform distribution, exhibiting superior performance compared to conventional CVD methods. Our analysis revealed two concurrent growth mechanisms on aluminum substrates: the tip-growth and base-growth modes, wherein the proportion of the base-growth mechanism exhibited significant temperature dependence. The present work establishes an innovative strategy for the low-temperature fabrication of high-performance CNT-based composite materials.

## 1. Introduction

Carbon nanotubes (CNTs) possess exceptional specific strength, elastic modulus, toughness, excellent wear resistance, and superior thermal and electrical conductivity, making them an ideal reinforcement phase for fabricating advanced composite materials [1,2,3]. CNTs can effectively enhance polymer, ceramic, and metal matrix composites [4,5,6]. Among these, CNTs are an ideal reinforcement for aluminum matrix composites. Krishna [7] conducted research on Al-CNT materials for thermal interface materials. Al-CNT composites were attempted for use in filler metals [8], and after welding, the tensile strength and microhardness of the weld metal were significantly improved. The addition of CNTs can markedly improve the mechanical properties of aluminum-based materials [9]. However, the reinforcing effect of CNTs as a reinforcement in matrix materials is influenced by various factors.

To improve the reinforcement and toughening effects of CNTs on matrix materials, the primary challenge lies in addressing the difficulty of achieving uniform dispersion of CNTs within the matrix [10,11,12]. The incorporation of CNTs into matrix powders typically involves two approaches: ex situ addition and in situ synthesis. In the ex situ method, mechanical ball milling [13] is conventionally employed to blend CNTs with matrix powders. However, due to the strong van der Waals interactions between CNTs, direct ball milling often fails to achieve uniform dispersion within the matrix [14,15]. While high-energy ball milling can enhance CNT dispersion of CNTs, extended processing times can induce substantial structural defects, compromising their reinforcement and toughening efficacy [16]. The in situ synthesis method typically utilizes chemical vapor deposition (CVD) [17] to grow CNTs on matrix powders spread in the reaction zone. CVD is a versatile technology with broad applicability, capable of preparing high-quality thin films, particularly in the realms of inorganic two-dimensional materials (e.g., graphene) and polymer films (e.g., semiconductor polymer films) [18,19]. CVD offers advantages in CNT dispersion compared to ball milling but exhibits inherent limitations. Traditional CVD employs a fixed-bed reactor, where powder particles are maintained in a static state. This restricts sufficient exposure of internal particles to carbon precursor gases, resulting in non-uniform CNT deposition on the matrix powder. Additionally, contact points between particles and between particles and reactor walls further exacerbate unevenness in CNT growth across particle surfaces. Due to these dispersion deficiencies inherent to traditional CVD, their capability for the large-scale production of CNT composite powders is limited [20]. Fluidized bed chemical vapor deposition (FBCVD) can overcome these limitations, but it requires stringent process control and faces challenges in synthesizing CNTs on powders with particle sizes below 20 μm [21,22,23].

To overcome the limitations of current CVD processes for in situ CNT synthesis, this study employs a novel rotating chemical vapor deposition (R-CVD) process. Catalyst-loaded matrix powders are placed in a furnace tube equipped with internal baffles. During the CVD process, the furnace tube rotates at a controlled speed while the baffles continuously lift and disperse the powders to ensure full exposure to reactive gases. This achieves complete catalyst reduction and uniform CNT growth on the powder surfaces. The rotation of the reactor enhances sample dispersion and mitigates sintering agglomeration of the matrix powder, especially for low-melting-point alloy powders, during the CVD process. Rotating reactors are not novel in CVD [24,25,26], which ensures complete contact between the surface of the powder particles and the reactive gas, thereby promoting uniform product deposition. This method is independent of powder particle size and offers improved process stability with a simpler apparatus [27]. Rotary reactors have also been employed for the large-scale synthesis of CNTs [28]. The R-CVD method has been applied in the preparation of coated powders, with the synthesized powders exhibiting excellent uniformity and dispersion [24,25]. This technique also demonstrates significant advantages in the fabrication of nanoparticle composite powders [29], enabling the synthesis of nanocomposite powders that are challenging to distribute uniformly via mechanical processing, thereby achieving superior dispersion. Furthermore, the versatility of R-CVD has been validated in the large-scale production of metal nanoparticles [23,30]. The direct synthesis of CNTs by the R-CVD process has limited applications. In the previous study of our group, the effect of R-CVD process parameters on CNT preparation was investigated by using alumina as the matrix powder, and CNT composite powders with more uniform distribution and higher yield were successfully synthesized at a lower reaction temperature with a higher purity of CNTs [1]. The synthesis temperature for preparing CNTs via CVD typically ranges from 600 to 1100 °C [31]. Lower synthesis temperatures can reduce energy consumption and enable CNT deposition on a wider variety of substrate materials, such as low-melting-point metals like aluminum. Additionally, applications of CNTs in flexible electronics, sensors, and integrated circuit interconnects require lower growth temperatures (25–500 °C) [31]. However, synthesis at lower temperatures results in the reduced catalytic cracking efficiency of carbon precursors, imposing limitations on the selection of both carbon sources and catalysts. Moreover, lower synthesis temperatures may affect CNT growth mechanisms on low-melting-point substrates. Taking aluminum as an example, variations in temperature lead to differences in the degree of Al softening, which in turn alters the bonding strength between Al and catalyst particles. These variations ultimately influence the CNT growth mechanism.

In this paper, we intend to use micro-sized pure aluminum powder as the matrix powder and systematically study the effects of synthesis temperature, synthesis time, catalyst content, carrier gas ratio, and furnace tube rotation speed on the CNT yield and structure of the R-CVD process. The aim is to provide a reference basis for the homogeneous deposition of CNTs in alloy matrix powders.

## 2. Materials and Methods

### 2.1. Synthesis of Al-CNTs Composite Powder

**Figure 1:**
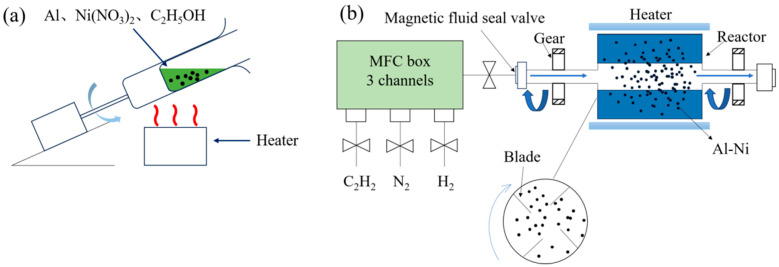
(**a**) Schematic of the mixing device. (**b**) Schematic of the rotary chemical vapor deposition (RCVD) apparatus.

Figure 1 shows a schematic diagram of a mixing device for loading catalysts on substrate Al powders and an R-CVD device for in situ synthesizing CNTs.

A specific Al powder (1–4 μm, analytical grade, Luxi Xiangchuang High-Performance Alloy Materials Factory, Luxi County, Xiangxi Autonomous Prefecture, China) and Ni(NO_3_)_2_·6H_2_O (analytical grade, ≥98.0%, Sinopharm Chemical Reagent Co., Ltd., Shanghai, China) ratio was mixed in anhydrous ethanol (analytical grade, ≥99.7%, Tianjin Fuyu Fine Chemical Co., Ltd., Tianjin, China). The mixture was heated using the mixing device (Figure 1a) until complete solvent evaporation. The resulting Al-Ni(NO_3_)_2_·6H_2_O composite powder was vacuum-dried in an oven at 100 °C for 8 h to ensure thorough solvent removal. The dried sample was ground and placed in the heating zone of a rotary tube furnace. Under N_2_ gas with a 120 mL/min flow rate, the temperature was raised to 400 °C and maintained for 120 min to calcine the powder, yielding Al-NiO. Subsequently, a mixed gas of N_2_ (240 mL/min) and H_2_ (120 mL/min) was introduced at 400 °C for 120 min to reduce NiO to Ni, producing Al-Ni fully. In previous studies [1], a furnace tube rotation speed of 7 r/min yielded optimal experimental results. Thus, throughout this process, the furnace rotation speed was fixed at 7 r/min. Finally, CNT synthesis optimization experiments were conducted by introducing C_2_H_2_/N_2_ gas mixtures (the total gas flow rate was set to 480 mL/min) under varying parameters: synthesis temperature, synthesis time, furnace rotation speed, and gas ratio of C_2_H_2_/N_2_ (all gases used in this work were 99.99% pure). The volume of the CNT growth zone was approximately 212,874 mm^3^. The design of experimental parameters referenced previous work by the research group [1].

### 2.2. Characterization Techniques

The morphology of the Al-CNTs composite powder was examined using a scanning electron microscope (ZEISS Sigma 300, Oberkochen, Germany). The microstructure of the powder at ultrahigh magnification was analyzed by TEM (JEOL JEM F200, Tokyo, Japan). A Raman microscope (Thermo Scientific DXR3, Waltham, MA, USA) was utilized to characterize the graphitic crystallinity of CNTs with an excitation wavelength of 532 nm. X-ray diffraction (Rigaku MiniFlex600, Tokyo, Japan) was employed to analyze the phase structure of the composite powder. To evaluate the carbon conversion efficiency of the R-CVD process, the CNT yield was calculated using the following equation [1]:

(1) $$CNT\;yields=\frac{M_{1}-M_{2}}{M_{2}}\times 100\%$$

M_1_ is synthesized CNT composite powder (g), and M_2_ is the Al-Ni catalyst (g) mass.

## 3. Results

### 3.1. Al-Ni Catalyst Powder

**Figure 2:**
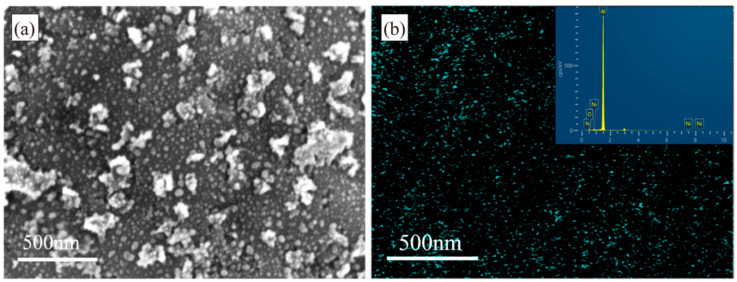
(**a**) SEM image of Al-Ni composite powder with 2.0 wt.% Ni content. (**b**) Ni elemental mapping.

The Al-Ni catalyst composite powder obtained after 120 min of reduction was characterized by SEM and elemental analysis, with the results presented in Figure 2. Figure 2a reveals numerous small particles on the Al surface. To determine the content and distribution of Ni on the Al surface, elemental mapping was performed, as shown in Figure 2b.

Based on Figure 2a, the Ni particle size distribution ranges from approximately 10 to 30 nm, with many smaller particles, some even below 10 nm. Meanwhile, some larger particles exceed 40 nm, reaching approximately 45 nm. Figure 2b indicates a uniform distribution of Ni elements on the Al surface, with a total Ni content of 2.24 wt.%, consistent with the anticipated results.

### 3.2. Synthesis of CNTs with Different Catalyst Contents

**Figure 3:**
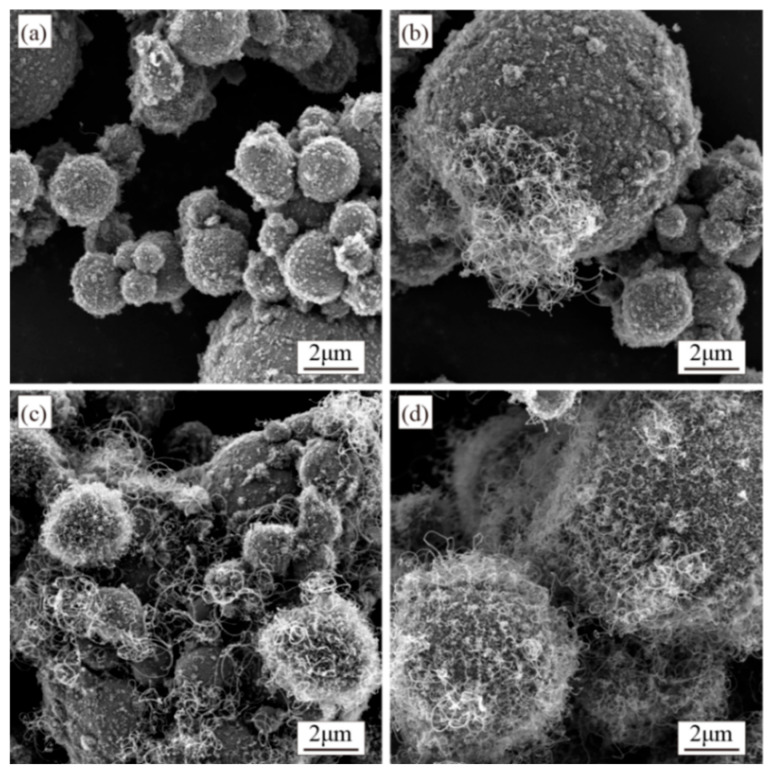
SEM images of CNTs synthesized at catalyst contents of (**a**) 0.5 wt.%, (**b**) 1.0 wt.%, (**c**) 1.5 wt.%, and (**d**) 2.0 wt.%.

The catalyst content significantly influences the product characteristics [32,33,34]. Using Ni as the catalyst, this study investigated CNT growth at Ni mass fractions of 0.5, 1.0, 1.5, and 2.0 wt.%. Based on preliminary optimization experiments [1], other parameters were fixed: synthesis temperature at 500 °C, growth time at 60 min, furnace tube rotation speed at 7 r/min, and a C_2_H_2_/N_2_ flow ratio of 1:7 (60 mL/min:420 mL/min). Morphological images of CNTs synthesized with different Ni contents are shown in Figure 3.

Figure 3a demonstrates that at a catalyst content of 0.5 wt.%, almost no CNTs were observed in the composite powder, with rod-shaped and flocculent carbon species formed instead. As the catalyst content increased, CNT generation gradually rose. At 1.0 wt.% and 1.5 wt.%, both the quantity and diameter of CNTs increased, though rod-like and amorphous carbon impurities persisted. CNTs exhibited uneven distribution on the Al surface at 1.0 wt.%, leaving many regions uncovered. This inhomogeneity improved at 1.5 wt.% but remained evident, with partial Al particles still lacking full CNT coverage at 2.0 wt.%, and CNTs displayed uniform diameter distribution and achieved homogeneous surface coverage across all Al particles.

**Figure 4:**
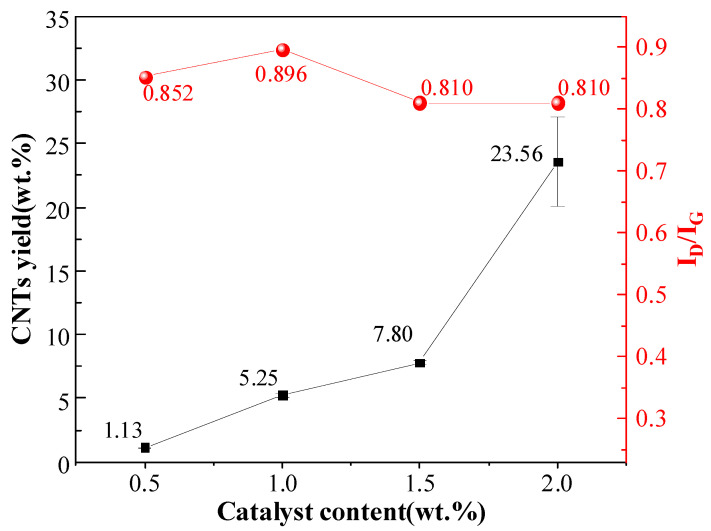
CNT yield and I_D_/I_G_ ratio for samples synthesized with different catalyst contents.

Raman spectroscopy analysis was performed on the composite powders to characterize the structural properties of CNTs synthesized via the R-CVD process. In Raman spectra, the D-band originates from amorphous carbon and impurities, with its intensity reflecting the relative content of defects, structural distortions, and amorphous phases in the sample, typically observed near 1300 cm^−1^. The G-band arises from the vibration of sp^2^-hybridized carbon atoms, indicating lattice integrity, and appears around 1600 cm^−1^. The synthesized samples’ crystallinity was evaluated using the D-band to G-band (I_D_/I_G_) intensity ratio, where a lower I_D_/I_G_ value corresponds to higher crystallinity. As shown in Figure 4, no significant differences in I_D_/I_G_ were observed among CNTs prepared with varying Ni contents, while the CNT yield exhibited an increasing trend with higher Ni catalyst loading.

### 3.3. Synthesis of CNTs with Different Synthesis Temperatures

**Figure 5:**
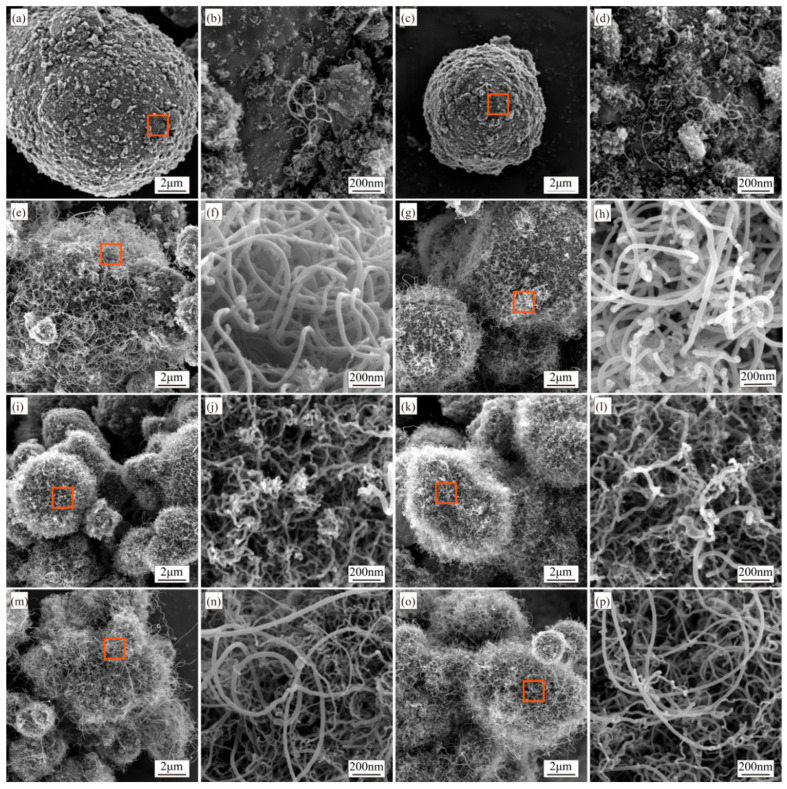
Al-CNTs composite powders were synthesized at varying temperatures, specifically (**a**,**b**) 440 °C, (**c**,**d**) 460 °C, (**e**,**f**) 480 °C, (**g**,**h**) 500 °C, (**i**,**j**) 520 °C, (**k**,**l**) 540 °C, (**m**,**n**) 600 °C, and (**o**,**p**) 620 °C. Orange boxes are further enlarged areas.

Synthesis temperature significantly impacts CNT formation. To investigate its influence and identify the optimal temperature for CNT synthesis via the R-CVD process, experiments were conducted under fixed parameters: a Ni content of 2.0 wt.%, a growth time of 60 min, a furnace rotation speed of 7 r/min, and a C_2_H_2_/N_2_ gas flow ratio of 1:7. CNTs were synthesized at eight temperatures: 440 °C, 460 °C, 480 °C, 500 °C, 520 °C, 540 °C, 600 °C, and 620 °C. Considering the melting point of Al (660 °C), this study wants to explore the growth of CNTs on Al substrates as the synthesis temperature approaches this value and to investigate the minimum synthesis temperature required for R-CVD-based CNT growth on Al. Based on previous research [1], a synthesis temperature range of 440–660 °C was selected. The morphology of the resulting Al-CNT composite powders was characterized by SEM, with results shown in Figure 5.

Figure 5a–d show negligible CNT formation at lower temperatures (440 °C and 460 °C), where rod-shaped and amorphous carbides cover the aluminum surfaces. The amount of CNTs increases progressively with temperature. At 480 °C and 500 °C, CNTs fully encapsulate Al particles, displaying clean and smooth walls (Figure 5e–h). Further temperature elevation to 520 °C and 540 °C (Figure 5i–l) induces aberrant CNT morphologies characterized by tortuous growth paths and surface irregularities. Approaching the melting point of Al (660 °C) at 600 °C and 620 °C, CNTs exhibit smooth surfaces and high uniform coverage across all Al particles, demonstrating temperature-dependent structural evolution (Figure 5m–p).

**Figure 6:**
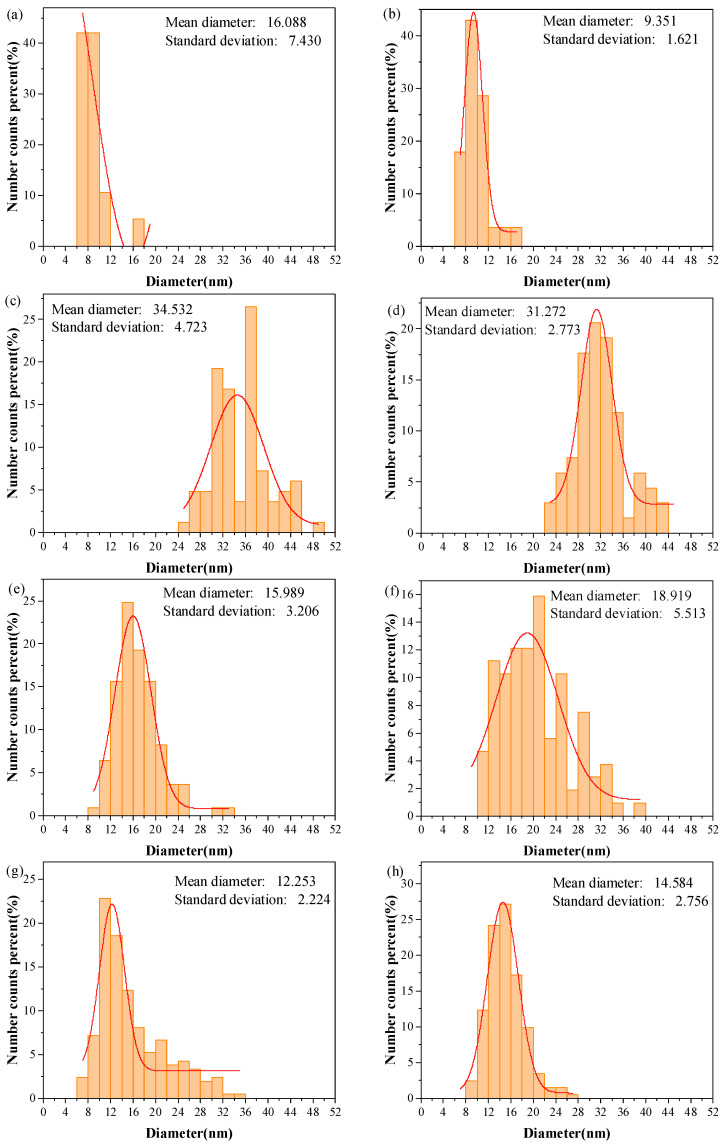
The diameter distribution of CNTs synthesized at different temperatures: (**a**) 440 °C, (**b**) 460 °C, (**c**) 480 °C, (**d**) 500 °C, (**e**) 520 °C, (**f**) 540 °C, (**g**) 600 °C, and (**h**) 620 °C. Curves are Gauss fits.

**Figure 7:**
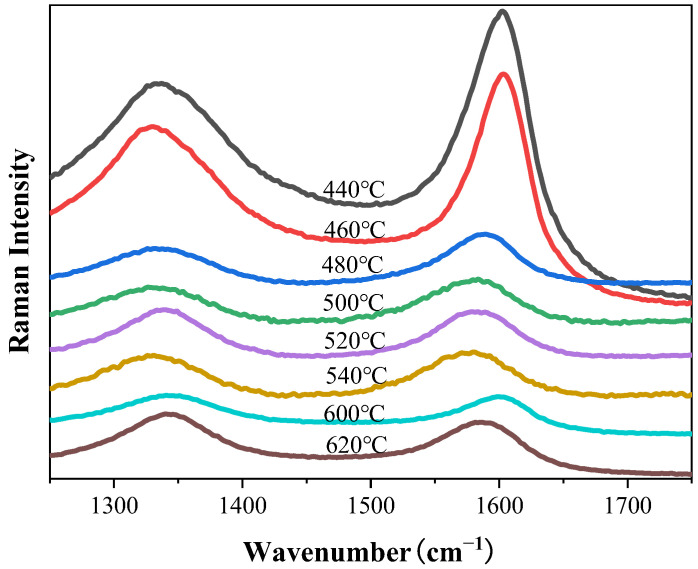
Raman spectra of composite powders synthesized at different temperatures.

Figure 6 displays the diameter distributions of CNTs synthesized at various temperatures. At 440 °C and 460 °C (Figure 5a–d), CNT formation is negligible, with no detectable CNTs observed. The scarcity of measurable diameter data at these temperatures invalidates further statistical analysis and thus excludes them from subsequent discussion. CNT diameters show significant growth for 480 °C and 500 °C, averaging approximately 35 nm and 31 nm, respectively. Notably, the diameter distribution at 500 °C exhibits a smaller standard deviation than 480 °C. At 520 °C and 540 °C, the mean diameters decrease to ~16 nm and ~19 nm, with the 540 °C sample displaying a slightly larger standard deviation. Further diameter reduction occurs at 600 °C and 620 °C (~12 nm and ~15 nm averages), where the narrowest standard deviations and most concentrated distributions are observed (Figure 6). Raman spectroscopy analysis of the composite powders synthesized across these temperatures is presented in Figure 7.

Data from Table 1 indicate that the sample synthesized at 480 °C exhibits the lowest peak intensity ratio (I_D_/I_G_), signifying the highest crystallinity. In the low-temperature range (440–460 °C), the I_D_/I_G_ ratio increases with the rising temperature. Within the mid-temperature range (480–540 °C), the ratio follows an upward trend with temperature except for a decrease at 540 °C. In the high-temperature range (600–620 °C), the ratio again increases with the temperature, demonstrating an overall ascending trend across the entire temperature spectrum. Additionally, the CNT yield initially rises with the temperature, then declines, and subsequently surges at 620 °C to reach the maximum value (Table 1). Specifically, the yield progressively increases up to 500 °C, decreases between 500 °C and 620 °C, and abruptly peaks at 620 °C.

**Figure 8:**
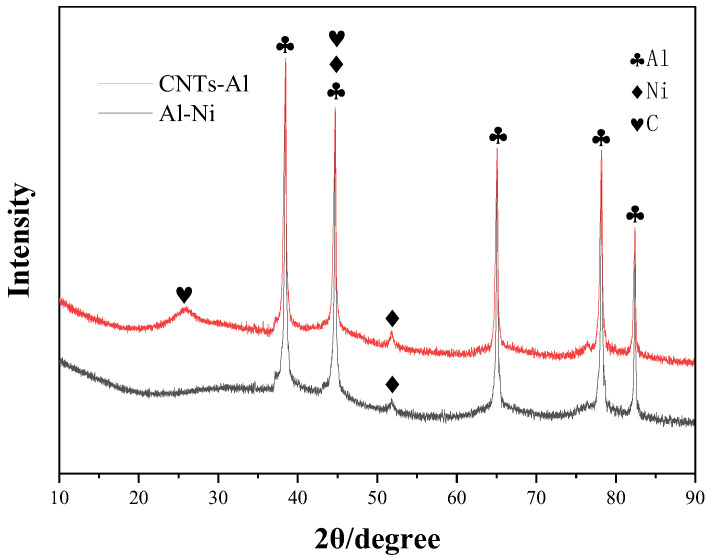
XRD patterns of the catalytic Al-Ni powder and the composite powder synthesized at 480 °C.

Figure 8 shows the XRD patterns of Al-Ni powder with 2.0 wt.% Ni content and CNTs synthesized via the R-CVD method at 480 °C. The results confirm the complete reduction in Ni in the catalyst composite powder after sequential processing steps (drying, calcination, and reduction), with no detectable NiO phases. Additionally, the diffraction peaks of CNTs are observed in the R-CVD synthesized sample, centered at 2θ ≈ 25.89°, corresponding to the characteristic (002) graphitic plane of CNTs.

### 3.4. Synthesis of CNTs with Different Rotation Speeds and Growth Times During Synthesis

**Figure 9:**
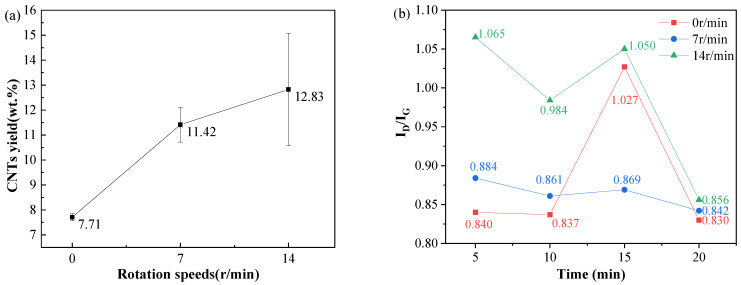
(**a**) CNT yield under different rotation speeds at a growth time of 5 min; (**b**) I_D_/I_G_ ratios at varying rotation speeds and growth times.

To mitigate the adverse effects of high CNT content on the subsequent processing of composite powders, synthesis duration was reduced under unchanged parameters (Ni content: 2.0 wt.%, temperature: 500 °C, C_2_H_2_/N_2_ gas flow ratio: 1:7) to obtain samples with lower CNT loading while maintaining favorable morphology. Growth times were set to 5, 10, 15, and 20 min. Three rotation speeds (0, 7, and 14 r/min (maximum rotation speed)) were applied during the synthesis stage to address potential insufficient gas–powder contact during shortened synthesis periods, with all other conditions held constant. The I_D_/I_G_ ratio from Raman analysis and CNT yield of the resulting composites are shown in Figure 9.

When the furnace tube remains stationary (0 r/min), the CNT yield is notably low (Figure 9a). Increasing the rotation speed enhances the yield accordingly (Figure 9a). Excluding minor anomalies, the I_D_/I_G_ intensity ratio exhibits an overall decreasing trend with a prolonged synthesis time, indicating progressive improvement in CNT crystallinity throughout the growth process.

**Figure 10:**
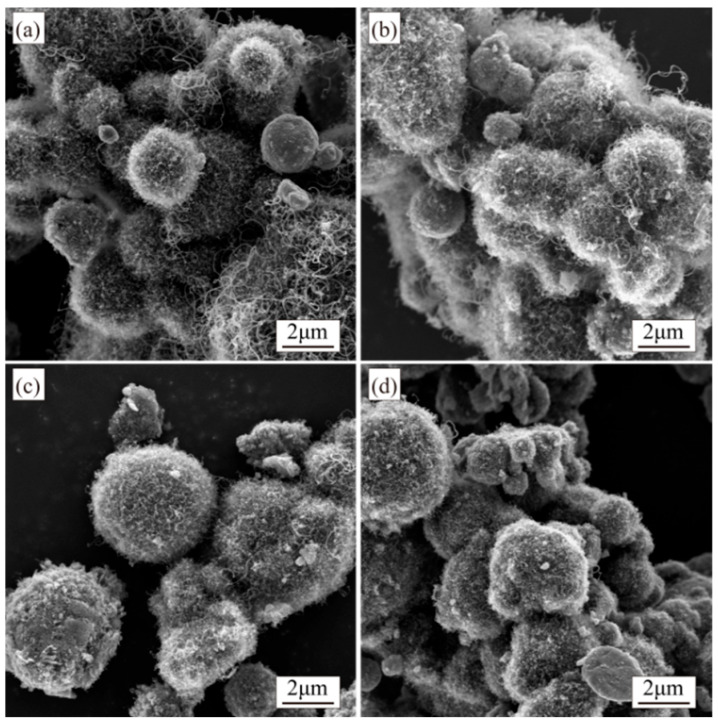
SEM images of composite powders synthesized at different reaction times under 7 r/min: (**a**) 5 min; (**b**) 10 min; (**c**) 15 min; (**d**) 20 min.

At a rotation speed of 7 r/min, CNTs synthesized across four different time intervals exhibit comparable morphological characteristics. The length and amount of CNTs gradually increase with prolonged growth while diameters remain small. Occasional larger-diameter CNTs are observed, though small-diameter CNTs dominate the population (Figure 10).

**Figure 11:**
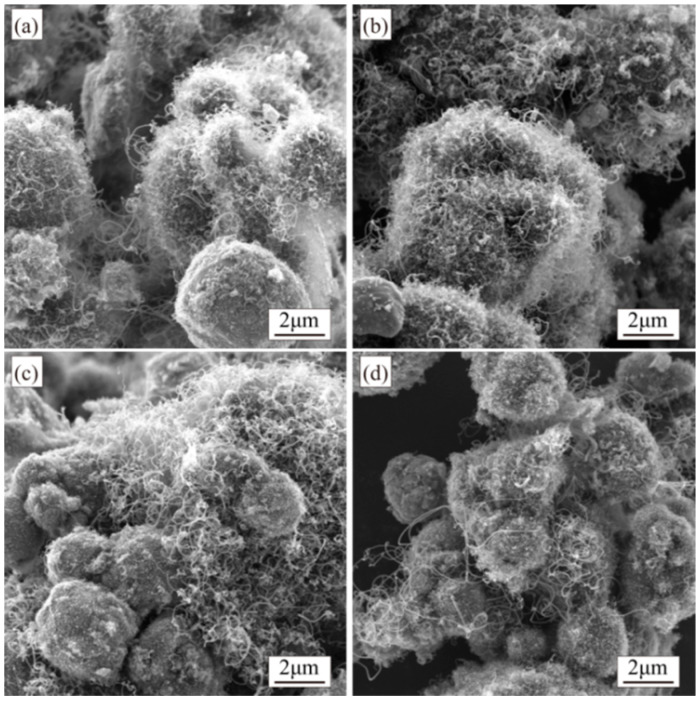
SEM images of composite powders synthesized at different reaction times under 14 r/min: (**a**) 5 min; (**b**) 10 min; (**c**) 15 min; (**d**) 20 min.

When the rotation speed is set to 14 r/min during synthesis, the quantity and length of CNTs increase compared to those synthesized at 7 r/min. Additionally, with an extended growth time under 14 r/min rotation, the CNTs’ quantity further rises. However, the complete coverage of Al particles remains unachieved, and the dispersion of CNTs across the Al surfaces remains inhomogeneous (Figure 11).

As shown in Figure 9b, at a rotation speed of 7 r/min, the I_D_/I_G_ ratios of samples synthesized across four time intervals show minimal variation, with the 20 min sample exhibiting a slightly lower value. At 14 r/min, a similar trend is observed: the 20 min sample achieves the lowest I_D_/I_G_ ratio, while the 5 min sample shows the highest value.

Across all three rotation speeds, the I_D_/I_G_ ratio exhibits a decreasing trend with prolonged growth time, though a sudden increase occurs at 15 min. The highest crystallinity is consistently achieved at 20 min. Increasing the furnace rotation speed notably elevates synthesized samples’ I_D_/I_G_ ratio, indicating that CNTs produced at 0 r/min possess the optimal crystallinity. Conversely, the CNT yield increases progressively with higher rotation speeds, as demonstrated in Figure 9a.

### 3.5. Synthesis of CNTs with Different C_2_H_2_ to N_2_ Gas Flow Ratios

**Figure 12:**
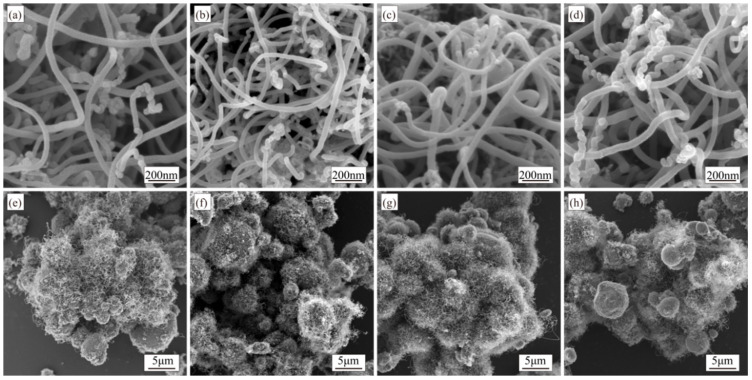
SEM images of CNTs synthesized under different C_2_H_2_/N_2_ gas flow ratios: (**a**,**e**) 1:5; (**b**,**f**) 1:7; (**c**,**g**) 1:9; (**d**,**h**) 1:11.

The ratio of carbon source gas to protective gas during the synthesis stage significantly influences CNT formation. In this experiment, using C_2_H_2_ as the carbon source and N_2_ as the protective gas, the synthesis of CNTs was systematically investigated at C_2_H_2_/N_2_ gas flow ratios of 1:5, 1:7, 1:9, and 1:11 (80 mL/min:400 mL/min, 60 mL/min:420 mL/min, 48 mL/min:432 mL/min, and 40 mL/min:440 mL/min) under fixed parameters: a Ni content of 2.0 wt.%, a synthesis temperature of 500 °C, a growth time of 60 min, and a furnace rotation speed of 7 r/min. Figure 12 presents SEM characterization results of CNTs synthesized under these varying gas flow ratios.

CNTs synthesized under different C_2_H_2_/N_2_ gas flow ratios exhibit similar morphologies with clean, smooth walls and abundant quantities, though their coverage uniformity on aluminum particles varies (Figure 12). Comparing Figure 12e–h, under the 1:5 ratio, a few Al surfaces display incomplete CNT coverage, while most particles exhibit CNT growth. Uniform CNT distribution and complete surface coverage are achieved at 1:7 and 1:9 ratios. However, reducing the ratio to 1:11 results in partial CNT-free areas on Al surfaces.

**Figure 13:**
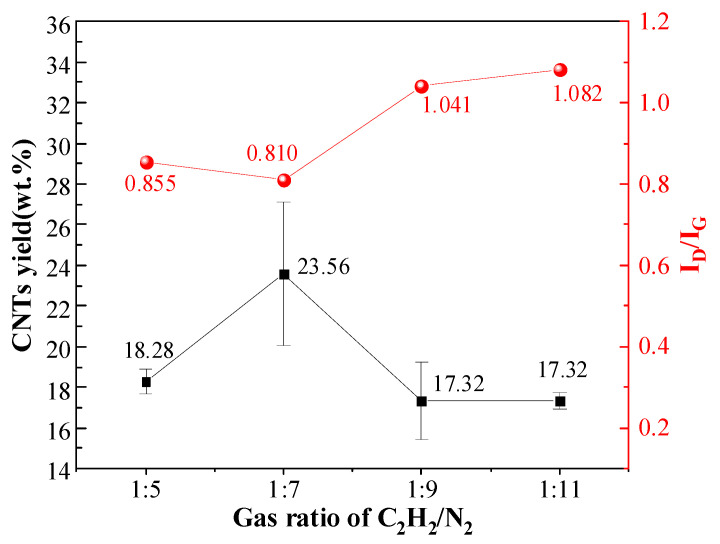
CNT yield and I_D_/I_G_ ratio under different C_2_H_2_/N_2_ gas flow ratios.

Figure 13 presents the yield and I_D_/I_G_ ratio of composite powders synthesized under different C_2_H_2_/N_2_ gas flow ratios. The I_D_/I_G_ ratio reaches its minimum value at a flow ratio of 1:7. As the gas flow ratio decreases from 1:5 to 1:7, the I_D_/I_G_ intensity ratio decreases, indicating improved CNT crystallinity. However, a further reduction in the C_2_H_2_ proportion gradually increases the ratio, suggesting a decline in crystallinity. Optimal crystallinity is achieved at the 1:7 ratio. The yield exhibits an initial increase followed by a decrease across the tested ratios (1:5 to 1:11). A significant yield drop occurs when the ratio shifts from 1:7 to 1:9. At the same time, minimal changes are observed from 1:9 to 1:11. The highest yield is attained at the 1:7 flow ratio.

## 4. Discussion

### 4.1. Growth Mechanism of CNTs

The process is hypothesized to involve the following stages for Ni nanoparticles’ catalytic formation of CNTs [34,35,36,37]: (1) C_2_H_2_ decomposition: acetylene molecules dissociate on the surface of Ni nanoparticles, generating carbon atoms; (2) carbon adsorption and carbide formation: the carbon atoms adsorb and deposit on the catalyst surface, forming carbides; (3) carbon diffusion: driven by combined concentration and temperature gradients, carbon atoms migrate into the interior of the catalyst particles; (4) supersaturation and CNT precipitation: once the dissolved carbon within the catalyst reaches supersaturation, CNTs nucleate and grow from the catalyst surface; (5) reaction termination: amorphous carbon fully encapsulates the catalyst surface, halting the reaction. If the interfacial interaction between the catalyst and substrate is weak, CNTs nucleate at the lower catalyst surface (the catalyst–substrate interface), exhibiting a tip-growth mode [35]. Conversely, CNTs nucleate on the upper catalyst surface when the interaction is strong, adopting a base-growth mode [38].

**Figure 14:**
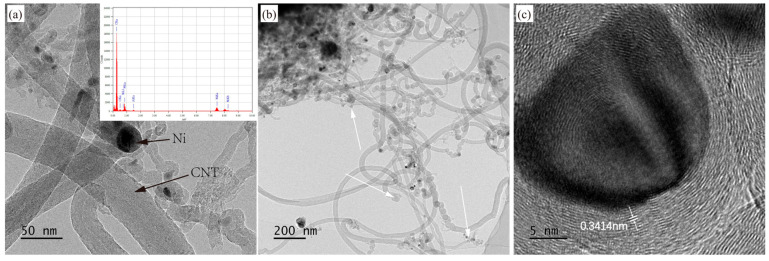
(**a**–**c**) are TEM images of the Al-CNT composite powders synthesized at 480 °C.

**Figure 15:**
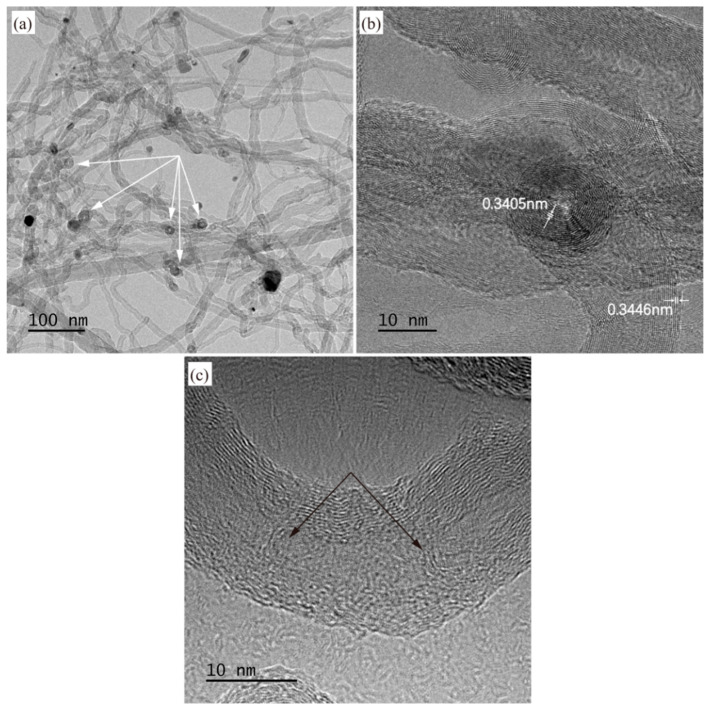
(**a**–**c**) are TEM images of the Al-CNT composite powders synthesized at 600 °C.

TEM analysis was performed on composite powders synthesized at 480 °C and 600 °C. For the 480 °C sample (Figure 14), the dark material encapsulating the tips of CNTs was identified via EDS analysis as carbon-coated Ni nanoparticles (Figure 14a). Additionally, some CNT tips were observed in an open-ended state without Ni nanoparticles (indicated by white arrows in Figure 14b), confirming the coexistence of tip-growth and base-growth mechanisms during CNT synthesis. Figure 14c reveals that the synthesized CNTs are multi-walled (MWCNTs), with an adjacent graphitic interlayer spacing of approximately 0.3414 nm, consistent with the ideal graphite interlayer distance. For the 600 °C sample (Figure 15), a higher proportion of CNT tips exhibited open-ended configurations lacking Ni nanoparticles (indicated by white arrow in Figure 15a). As shown in Figure 15b, the adjacent interlayer spacing at the open tips measures ~0.34 nm, while the wall spacing of CNTs is ~0.3446 nm, slightly larger than that of the 480 °C sample. This aligns with Raman results, indicating higher crystallinity in the 480 °C sample. Furthermore, minor graphitic interlayers within the CNT cores were observed (indicated by black arrows in Figure 15c), a phenomenon consistent with findings reported by Li et al. [39] and Yang et al. [40].

**Figure 16:**
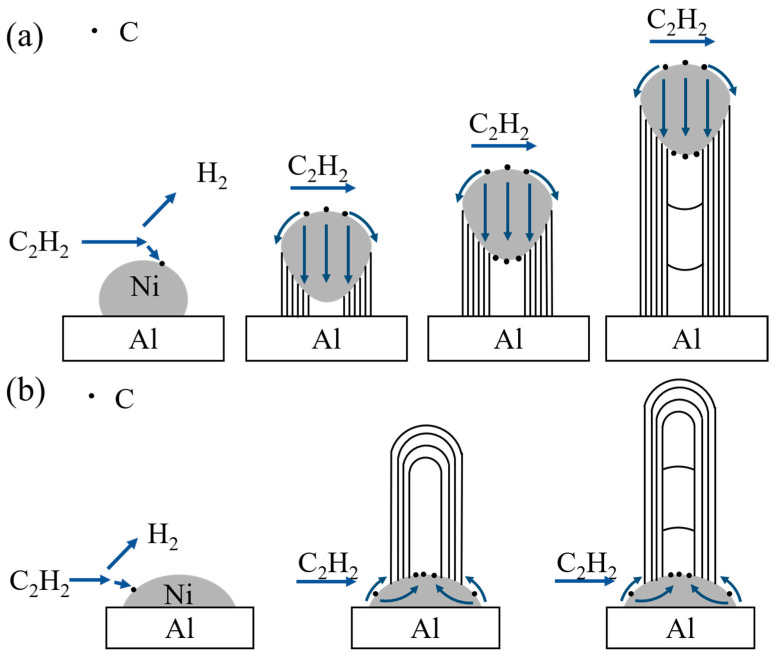
(**a**) Schematic of CNT tip-growth mode. (**b**) Schematic of CNT base-growth mode.

Under identical synthesis conditions, CNTs grown on an Al_2_O_3_ substrate predominantly follow a tip-growth mode [1]. In contrast, both tip-growth and base-growth modes coexist on an Al substrate, consistent with findings reported by Duan et al. [41]. Compared to the high melting point of Al_2_O_3_, Al’s lower melting point facilitates substrate softening near its melting temperature. This softening enhances the “pinning effect” on surface Ni nanoparticles, strengthening their interfacial bonding with the Al substrate and inhibiting detachment. Consequently, the base-growth mode becomes more prevalent. At elevated temperatures (600 °C), the increased prevalence of open-ended CNTs further confirms this mechanistic shift. Figure 16 illustrates the key mechanisms governing CNT growth on the Al substrate. Acetylene gas decomposes on the surface of nickel (Ni) nanoparticles, releasing C atoms that adsorb onto the Ni surface. These atoms subsequently diffuse and precipitate to form CNTs. For Ni particles with weak interfacial bonding to the substrate (Figure 16a), CNTs nucleate at the Ni–substrate interface, causing the Ni particles to lift and detach from the substrate. Acetylene decomposes, generating C atoms that diffuse across the Ni nanoparticles. Some C atoms continue to nucleate and grow CNTs, while others diffuse to the lower surface of the Ni particles, forming the graphitic core structure of the CNTs. Some Ni nanoparticles exhibit strong interfacial bonding with the substrate due to the “pinning effect” of the Al matrix, resisting detachment (Figure 16b). In such cases, CNTs nucleate and grow on the upper surface of the catalyst particles. Analogous to the tip-growth mode, a portion of carbon atoms diffusing to the upper surface of the Ni nanoparticles contributes to the formation of graphitic layers within the CNT core.

### 4.2. Effect of Catalyst Contents on CNT Synthesis

The reduced number of catalyst particles on the Al substrate results in fewer available reaction sites and lower catalytic activity at low catalyst content. This leads to the insufficient decomposition of C_2_H_2_ into carbon atoms, thereby hindering CNT synthesis. As demonstrated in the study by Lin et al. [32], lower Ni catalyst content requires higher initiation temperatures for CNT growth, further confirming the diminished catalytic activity at reduced catalyst loadings. Increasing the Ni content enhances the population of active catalyst particles, improving the efficiency of C_2_H_2_ decomposition and generating more carbon precursors. This promotes higher CNT yields (Figure 3) and improved crystallinity (Figure 4). When the Ni content is below 2.0 wt.%, the quantity and yield of CNTs are extremely low. However, increasing the Ni content to 2.0 wt.% significantly boosts the yield while achieving better crystallinity in the synthesized CNTs.

### 4.3. Effect of Synthesis Temperature on CNTs’ Synthesis

At a fixed Ni catalyst content, the synthesis temperature critically influences the catalytic activity of Ni. Low temperatures suppress Ni activity, while excessively high temperatures may induce catalyst deactivation [32,42]. Under low-temperature conditions (440 °C and 460 °C), the diminished Ni activity presents dual challenges: (1) Elevated activation energy for C_2_H_2_ decomposition: the limited thermal energy at low temperatures reduces the dissociation efficiency of C_2_H_2_, resulting in insufficient carbon atom generation to sustain CNTs growth. (2) Inhibited carbon diffusion: reduced concentration and temperature gradients within Ni nanoparticles slow the diffusion rate of carbon atoms. This promotes surface accumulation of amorphous carbon rather than graphitic CNT formation. These factors collectively lead to minimal CNT production at low temperatures, as evidenced by the sparse CNT populations shown in Figure 5 and quantified in Table 1. At moderate temperatures (480–540 °C), the catalytic activity of Ni significantly improves, and the elevated thermal energy sufficiently drives both C_2_H_2_ decomposition and CNT synthesis, resulting in higher CNT yield. At 480 °C, CNTs exhibit optimal crystallinity, suggesting that the rate of CNT formation surpasses amorphous carbon deposition. This implies that the diffusion rate of C atoms on the Ni surface and the catalytic synthesis rate of CNTs exceed the rates of C_2_H_2_ decomposition and carbon deposition at this temperature. When the temperature increases to 500 °C, accelerated C_2_H_2_ decomposition enhances carbon atom diffusion and CNT synthesis rates, further boosting CNT production. However, the rapid deposition of carbon atoms on the Ni surface leads to incomplete diffusion, causing residual carbon to form amorphous phases. Consequently, while the CNT yield increases at 500 °C, the crystallinity decreases due to competing amorphous carbon formation. When the temperature rises to 520 °C and 540 °C, the decomposition rate of C_2_H_2_ further accelerates. Excessive carbon atoms deposit on the Ni surface faster than they can be incorporated into CNTs, leading to the partial deactivation of Ni nanoparticles. Consequently, the CNT yield decreases with increasing temperature, and the crystallinity of the CNTs also deteriorates. In the high-temperature range (600 °C and 620 °C), the crystallinity of CNTs further decreases, indicating increased amorphous carbon formation. However, the yield rises significantly at 620 °C, suggesting enhanced carbon diffusion and C_2_H_2_ decomposition rates that promote CNT growth and amorphous carbon generation. Combined with the observed smaller CNT diameters at 620 °C, the “pinning effect” increases the density of Ni nanoparticles on the Al surface, enabling higher CNT production. Nevertheless, the reduced surface area of individual Ni nanoparticles accelerates carbon overcoating and catalyst deactivation, fostering amorphous carbon deposition. Under these dual effects, the 620 °C condition achieves the highest overall yield despite compromised crystallinity.

The diameter distribution of CNTs synthesized at different temperatures (Figure 6) reveals a gradual reduction in CNT diameter with increasing synthesis temperature, closely correlated with the size of catalyst particles [43,44]. Compared to CNTs synthesized at other temperatures, those formed at elevated temperatures exhibit smaller diameters, averaging ~15 nm. At 520–540 °C, the average diameter ranges between 15 and 20 nm, while at 480–500 °C, the average diameter increases to 30–35 nm. This phenomenon may arise from the “pinning effect” of the Al matrix. As the synthesis temperature approaches the melting point of Al, the softened Al matrix immobilizes Ni particles, suppressing their agglomeration. The closer the temperature is to the Al melting point, the more pronounced the pinning effect becomes. However, as the temperature increases, the agglomeration tendency of Ni nanoparticles intensifies. Although CNT diameters decrease at 520–540 °C, their distributions exhibit larger standard deviations, indicating broader size dispersity. The standard deviation minimizes at 600–620 °C, showing highly concentrated diameter distributions. This suggests that Ni atoms retain significant agglomeration tendencies during the R-CVD process under elevated temperatures and rotational dynamics, where temperature critically influences nanoparticle coalescence. Concurrently, the enhanced softening of the Al substrate at higher temperatures strengthens the “pinning effect” on Ni particles—this dual effect results in reduced CNT diameters and narrowed size distributions at near-melting temperatures.

In this study, CNTs with high crystallinity and abundant yield were successfully synthesized at 480 °C (Figure 5e), a temperature significantly lower than those reported in the prior literature (Table 2). This phenomenon can be attributed to the rotational dynamics of the R-CVD process, which enhances gas–powder contact efficiency. Improved interaction between carbon precursor gases and Ni catalyst particles increases carbon deposition on the Ni surface, while the intensified concentration gradient accelerates carbon diffusion into the Ni nanoparticles. These synergistic effects enable efficient CNT synthesis at reduced temperatures.

The deposition rate of CNTs was defined as the ratio of the mass of CNTs generated per unit of time to the mass of the pre-reaction Al-Ni catalyst. A comparison of the highest deposition rate achieved in this study with those reported in other works is presented in Table 3. This work achieved a relatively favorable CNT deposition rate under conditions of lower catalyst loading.

### 4.4. Effects of Rotation Speed and Growth Time During Synthesis on CNT Synthesis

**Figure 17:**
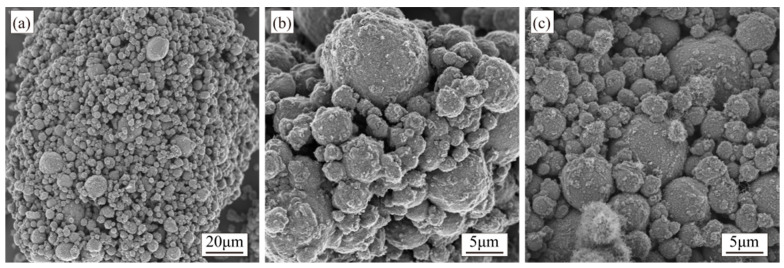
Aggregation at 0 r/min for (**a**) 5 min, (**b**) 10 min, and (**c**) 20 min.

During the synthesis stage, the introduced C_2_H_2_ gas is decomposed into carbon atoms via Ni catalysis. Ni nanoparticles require a certain amount of dissolved carbon atoms to initiate CNT nucleation. Under short synthesis durations, an insufficient amount of time for C atoms to diffuse into the Ni particle interior results in limited CNT formation. Extending the reaction time allows deeper carbon dissolution into the Ni matrix, facilitating the synthesis of higher quantities of structurally intact CNTs. Consequently, the I_D_/I_G_ intensity ratio decreases with prolonged synthesis time, reflecting improved crystallinity. Furthermore, increasing the rotation speed during synthesis enhances CNT yield. This is attributed to the improved gas–powder contact under rotational conditions, where even the interfaces between underlying powder layers and particle contact zones gain access to the carbon precursor gas [20]. Such enhanced interaction mitigates the agglomeration tendency of Al particles, enabling full surface exposure to the gas phase and thereby boosting yield. Consequently, CNTs synthesized at 14 r/min exhibit higher density and more uniform distribution across matrix particles (Figure 11), whereas static or low-speed conditions promote particle agglomeration (Figure 17). However, as the rotational speed increased, the I_D_/I_G_ ratio of the CNT composite powder also rose, likely due to enhanced amorphous carbon formation during synthesis. This could be explained by the improved contact between Ni catalyst nanoparticles and carbon precursor gases under high-speed rotation, which accelerates carbon atom generation. However, during shorter synthesis times, excess carbon atoms may not fully diffuse to CNT growth zones, leading to amorphous carbon accumulation on the catalyst surface. When the synthesis time was extended to 20 min, the I_D_/I_G_ ratios became more comparable, suggesting that prolonged rotation promotes carbon atom diffusion to CNT growth regions, reducing amorphous carbon defects.

### 4.5. Effects of C_2_H_2_/N_2_ Gas Flow Ratio on CNT Synthesis

The gas flow ratio regulates CNT growth by controlling carbon atom generation. At a fixed total gas flow rate, lowering the C_2_H_2_/N_2_ ratio reduces the C_2_H_2_ supply, thus decreasing carbon atom production. Fewer carbon atoms are available when the ratio drops from 1:7 to 1:11, resulting in reduced CNT quantities and degraded crystallinity (lower yield and higher I_D_/I_G_ ratios). When the C_2_H_2_/N_2_ gas flow ratio decreases from 1:5 to 1:7, the CNT yield and crystallinity increase. This is because, at a 1:5 ratio, the carbon atom generation rate exceeds the diffusion rate. Excess carbon atoms deposit as amorphous carbon on the Ni nanoparticle surfaces, covering active catalytic sites and deactivating the Ni particles, thereby reducing CNT synthesis. When the flow ratio decreases to 1:7, the reduced C_2_H_2_ supply balances carbon generation and diffusion rates. This minimizes amorphous carbon formation, preserves Ni catalytic activity, and enhances CNT yield and crystallinity. When the C_2_H_2_/H_2_ gas flow ratio decreases, the decomposition rate of C_2_H_2_ to generate carbon atoms decreases further. The C atom generation rate becomes lower than the diffusion rate. These factors hinder CNT synthesis, as insufficient carbon atoms sustain CNT growth. Consequently, depositing sufficient C atoms on the Ni surface requires a longer period of time. In addition to the impact of the carbon precursor gas flow rate, the flow rate of N_2_ also significantly affects CNT synthesis. Increasing the N_2_ flow rate reduces the concentration of C_2_H_2_ in the reaction zone, lowering the C_2_H_2_ content near the Ni surface. This helps avoid excessive carbon atom adsorption on the Ni surface, removes excess carbon precursor gas, and provides sufficient time for carbon atoms to diffuse into the catalyst interior, thereby reducing surface amorphous carbon formation and delaying catalyst particle deactivation. However, excessively high N_2_ flow rates may overly dilute the C_2_H_2_ concentration near the Ni surface, while N_2_ molecules might adsorb onto the Ni surface, occupying active reaction sites and hindering carbon atom generation, ultimately impairing CNT growth.

## 5. Conclusions

This study prepared Al matrix composite powders with Ni catalysts via the impregnation method, followed by the in situ synthesis of CNTs on the Al substrate using R-CVD. The effects of catalyst content, synthesis temperature, rotation speed, growth time, and C_2_H_2_/N_2_ gas flow ratio on the morphology, structure, and yield of CNTs were systematically investigated. The growth mechanisms of CNTs on the Al substrate were also elucidated. The quality of synthesized CNTs was evaluated through SEM, Raman spectroscopy, and TEM. Key conclusions are as follows:

1. Tip-growth and base-growth mechanisms coexist during CNT synthesis on the Al substrate.
2. Excessive catalyst loading hinders subsequent processing, while insufficient loading impedes CNT growth. A Ni content of 2.0 wt.% achieves optimal CNT morphology and purity.
3. Low temperatures reduce carbon diffusion rates, limiting yield, while excessive temperatures promote amorphous carbon formation. A synthesis temperature of 500 °C balances yield and CNT purity.
4. Near the melting point of Al, CNTs exhibit smaller diameters and narrower size distributions but significantly reduced crystallinity.
5. Prolonged synthesis times enhance CNT length and density, while higher rotation speeds increase yield but degrade crystallinity.
6. Short synthesis results in non-uniform CNT distributions, elevated defect densities, and amorphous carbon impurities. To achieve high-quality, uniformly dispersed CNTs, sufficient synthesis duration is required.
7. Reducing the C_2_H_2_/N_2_ flow ratio initially increases CNT yield and crystallinity, peaking at 1:7. Further reduction causes carbon starvation, sharply decreasing both parameters.

## Figures and Tables

**Table 1:** CNT yield and I_D_/I_G_ ratio of composite powders synthesized at different temperatures.

Temperature (℃)	CNT Yield (wt.%)	I_D_/I_G_
**440**	13.02 ± 0.47	0.750
**460**	14.02 ± 0.30	0.774
**480**	16.17 ± 1.77	0.708
**500**	23.56 ± 3.54	0.810
**520**	21.85 ± 1.48	1.047
**540**	19.22 ± 0.87	0.919
**600**	18.74 ± 0.54	1.027
**620**	23.99 ± 4.29	1.154

**Table 2:** A list of synthesis temperatures was obtained from the other literature.

Matrix	Catalyst	Carbon Source Gas	Temperature (°C)	References
Al	Ni	C_2_H_2_	480	This work
ZrB_2_	Ni	C_2_H_2_	1050	[32]
TiB_2_	Ni/Y_2_O_3_	CH_4_, C_2_H_2_, CO	800	[45]
CuCrZrY	Cr	C_2_H_4_	800	[46]
TC_4_	Fe	CH_4_	600	[47]
Aluminum Foil	Ni	C_2_H_2_	645	[48]

**Table 3:** List of deposition rates from the literature.

Catalyst Loading (wt.%)	CNT Deposition Rate (g/(g·h))	Reference
2.0	0.2399	This work
2.0	0.2197	[1]
0.3	0.0300	[49]
14.0	8.3700	[50]

## Data Availability

The original contributions presented in this study are included in the article. Further inquiries can be directed to the corresponding author.
